# Influence of silane type and application time on the bond strength to leucite reinforced ceramics

**DOI:** 10.1080/07853890.2021.1897360

**Published:** 2021-09-28

**Authors:** Anastásia Rita, João Reis, Inês Caetano Santos, António H. S. Delgado, João Rua, Luís Proença, José João Mendes

**Affiliations:** a Instituto Universitário Egas Moniz, Almada, Portugal; b Centro de Investigação Interdisciplinar Egas Moniz (CiiEM), Almada, Portugal

## Abstract

**Introduction:**

All-ceramic restorations currently dominate the market of indirect restorative materials due to their biocompatibility, longevity and superior aesthetics [1]. Silica-based ceramics, such as leucite, carry the advantage of being receptive to surface treatments, making them bondable to tooth substrates [2]. However, a standardised application protocol regarding silane coupling agents is lacking. Such step is critical to ensure durability of the restoration placed *in situ*. Post-etch cleaning and silane application have been proven to increase bond strength, however, this step varies for each material [3]. The aim of this research was to assess the influence of different types of silane coupling agents and respective application times on the bond strength of the ceramic-resin interface.

**Materials and methods:**

Ten leucite reinforced glass ceramic blocks (IPS Empress CAD LT BL4/C 14) were divided into equal halves. Of the samples obtained, 6 were randomly divided into three groups according to the silane used: **G1** BIS-Silane (Bisco, Schaumburg, IL, USA); **G2** ESPE Sil Silane Coupling Agent (3 M ESPE AG, Seefeld, Germany); **G3** Monobond Plus (Ivoclar-Vivadent, Schaan, Liechtenstein). Each was then divided into two subgroups, according to the surface conditioning time: **T1** (1 min.) or **T2** (5 min.). Each block was acid etched (HF 9.5% − 1 min), post-etching cleaned (OPA 37.5% − 1 min; ultrasonication − 2 min.) and silanized. Heat treatment was carried out at 100 °C (1 min.). Then a thin layer of Optibond FL (Kerr) adhesive was applied and each block was adhered to pre-heated resin at 55 °C. The samples were light cured for 40 s on each side (1200 mW/cm^2^). Samples were sectioned into microspecimens (1 ± 0.2 mm^2^) that were subjected to aging (10,000 thermocycles − 5 and 55 °C). The microspecimens were tested in tension at a crosshead speed of 0.5 mm/min, until they debonded. Data analysis was carried out by a two-way ANOVA, at a significance level of 5%.

**Results:**

The group featuring BIS-Silane with longer application time (G1T2) presented a mean µTBS value (32.4 ± 19.6 MPa) significantly higher to all other groups (*p* < .001). Monobond Plus registered the lowest mean µTBS value (G3T1 − 18.5 ± 7.3 MPa) and (G3T2 − 17.3 ± 5.8 MPa). The type of silane coupling agent has shown to have a significant influence on the microtensile bond strength (*p* = .001; *η*^2^=0.16).

**Discussion and conclusions:**

Some authors have previously suggested that silanization could benefit from longer application times, but seldom research has been found featuring this variation protocol [3,4]. Silanes require a hydrolysation process in order to establish chemical bonds. Two-bottle systems show the highest bond strength results and may benefit from longer application times. The addition of 10-MDP seems to have no significant advantage over traditional silane coupling agents.
